# Medical Students’ Attitudes Towards Research Participation After the United States Medical Licensing Examination Step 1 Scoring Change

**DOI:** 10.1007/s40670-025-02508-3

**Published:** 2025-09-19

**Authors:** Danxun Li, Nina Li, Curt Bay, Uzoma Ikonne

**Affiliations:** 1 Eastern Virginia Medical School, Old Dominion University, Norfolk, VA 23507 USA; 2 Department of Interdisciplinary Health Sciences, Arizona School of Health Sciences, A.T. Still University, 5850 E. Still Circle, Mesa, AZ 85206 USA; 3 Department of Biomedical and Translational Sciences, Eastern Virginia Medical School, Old Dominion University, 700 W. Olney Rd, Norfolk, VA 23507 USA; 4 Fine Family Academy of Educators, Eastern Virginia Medical School, Old Dominion University, Norfolk, VA 23507 USA

**Keywords:** Medical student sesearch, Undergraduate medical education and USMLE step 1

## Abstract

**Background:**

The recent transition of scoring for the United States Medical Licensing Examination (USMLE) Step 1 exam from a numerical grading system to pass/fail system has significant implications for residency applicants. It is anticipated that research productivity will gain greater importance in the evaluation process. To understand how medical institutions can best support students, we seek to understand medical students’ motivations and experience conducting research in medical school after the USMLE Step 1 scoring change.

**Methods:**

This is a mixed-methods, cross-sectional study that examines the motivations and barriers that medical students face in conducting academic or clinical research. A survey study based on a modified questionnaire for medical students was distributed to second-year medical students at a single institution. Mann–Whitney tests were used to analyze differences in attitudes across various subgroups and Pearson correlations quantified the strength of relationships. A thematic analysis was used to examine the qualitative data set.

**Results:**

68 students completed the survey, 35 male and 32 female, and one failed to select an option. 39 students (57.3%) indicated that the USMLE Step 1 transition to pass/fail has influenced their attitudes and beliefs about conducting research in medical school. The most frequently endorsed motivation for medical student research participation was to be more competitive for residency applications [(65/68), 95.6%]. The most frequently endorsed barrier was lack of time [(59/68), 86.8%]. Research participation is correlated with research output (*r* = 0.49, *p* < 0.001). Competitiveness of specialty of interest was correlated with research participation (*r* = 0.36, *p* < 0.01) but not research output (*p* > 0.05). There is no difference in research output, participation, or attitudes between genders (*p* > 0.05).

**Conclusions:**

Medical students perceive research participation and productivity as an increasingly important differentiating factor in competitive residency applications as USMLE Step 1 transitions to pass/fail. However, lack of time to engage in research poses a barrier for many students. Efforts that support student endeavors in academic or clinical research are a crucial aspect of the medical school curriculum and should focus on streamlining student research participation as the preclinical curriculum shortens across medical schools.

**Supplementary Information:**

The online version contains supplementary material available at 10.1007/s40670-025-02508-3.

## Background

The recent transition of the scoring for the United States Medical Licensing Examination (USMLE) STEP 1 exam from a numerical grading system to a pass/fail has significant implications for residency applicants [1]. The purpose of the USMLE Step 1 is to serve as a licensing exam; however, in a 2020 National Residency Match Program (NRMP) survey of program directors, the USMLE Step 1 score was the most commonly cited factor in selecting applicants for interviews [2, 3]. One of the aims of the scoring change was to encourage a more holistic residency selection process, with increased consideration of other standardized evaluation tools and the individual attributes of applicants [1]. However, the growing number of residency applications presents challenges for holistic evaluations, necessitating alternative methods for distinguishing applicants.

The impact of this scoring transition remains uncertain, and it is unlikely to impact all medical students equally. It is anticipated that there will be an increased emphasis on factors such as institutional prestige, USMLE Step 2 score, and clerkship evaluations [4, 5]. Additionally, research productivity is anticipated to play a significant role in applicant evaluation, as it provides students with opportunities to network, engage, and learn more about a specific field. Furthermore, students can develop highly sought-after skills in critical thinking, communicate complicated information clearly, and work in a team setting. Research accomplishments have already been used as a differentiating factor in highly competitive specialties, with previous studies demonstrating that matched applicants tend to have a greater number of research accomplishments than unmatched applicants [6–8].

Research experience, publications, and presentations may play a greater role in the residency match process; therefore, medical schools should evaluate their infrastructure and resources for supporting research among their students [9, 10]. An effective research environment includes opportunities for research presentations, dedicated time for research, and financial support for conference travel. While the NRMP provides quantitative data on residency applicants’ publications, there is a lack of studies focusing on the research experiences of medical students [11]. To effectively support students in research, it is crucial to understand their attitudes, motivations, and experiences. This study aims to explore medical students’ participation in research after the USMLE Step 1 scoring change.

## Methods

In this investigation, we aimed to gain insights into the attitudes, motivations, and experiences of medical students engaged in research at Eastern Virginia Medical School (EVMS), an MD-granting medical school in the United States. The Eastern Virginia Medical School Institutional Review Board approved the study, IRB # 23–09-XX-0220.

### Survey Design

An electronic survey was generated using REDCap, consisting of multiple-choice and short-response questions (Appendix). The questionnaire was adapted from an earlier study, one of the few published surveying medical students on their motivations for conducting research in medical school [12]. Most questionnaire questions related to study objectives were presented in Likert-scale format. Students were asked to select their level of agreement to a list of factors that serve as motivators, and a list of factors that serve as barriers to conducting research in medical school. In addition, students were asked to select the top three factors that serve as motivators and barriers. The questionnaire asked about student demographic information, including gender, age, ethnicity, and highest degree attained. Additional questions inquired about students’ specialty of interest and asked them to describe the competitiveness of their specialty of interest. Students were asked to indicate the number of research studies in which they have participated, and the number of abstracts, presentations, or publications they have produced. The questionnaire also included open-ended questions for students to provide written feedback concerning motivators for research, barriers for research and the role of research in their future career.

### Recruitment

In the fall of 2023, pre-clerkship medical students from the MD class of 2026 were invited by the principal investigator to complete the questionnaire. This class was chosen as one of the first cohorts to enter medical school with the knowledge that Step 1 will be pass/fail, giving them sufficient time to plan for residency applications with the score change. An email containing a link to the questionnaire was subsequently sent. Student participation was voluntary and anonymous, and they were informed that completion would not impact their academic standing. Two follow-up emails were sent as a reminder to participate two weeks and four weeks following the initial email invitation.

### Statistical Analytics

Counts and percentages are provided as summary descriptive statistics. Mann–Whitney tests were used to analyze differences in attitude and performance across various subgroups, and Spearman correlations quantified the strength of relationships between perceived specialty competitiveness and research activity. Effect sizes (ES) are provided for Mann–Whitney tests. The sample responding to the survey (n = 68) should yield a confidence level of approximately 95% ± 10% for students in the target cohort (n = 150). The 68 respondents are sufficient to identify a medium effect size (r between 0.3 and 0.5) for Mann–Whitney and correlational tests with 80% power, alpha = 0.05, two-tailed. An alpha of 0.05 (two-tailed) was the criterion for statistical significance. SPSS ver. 28 (IBM Corp., Armonk NY) was used for all analyses.

### Qualitative Analysis

Open coding [13] was used to analyze qualitative survey data generated from student responses to open-ended questions. At the initiation of the coding process, two investigators, N.L. and U.I., independently reviewed the student responses to the open-ended questions, made annotations based on the student responses, and identified themes that emerged from the data. After the investigators finished independently coding the data, they met together to discuss the themes they identified.

## Results

### Participants

A total of 69 surveys were completed, of which 68 were included in the final analysis. A duplicate response (n = 1) was excluded. The final sample of 68 students represents 45.3% of the second-year pre-clerkship class. Participants included 35 males (51.5%), 32 females (47.1%), and 1 student who did not indicate their gender (1.47%) (Table 1). Most respondents were 22–25 years old (52.9%).

**Table 1:** Demographics

Demographic Characteristic	No. (%)
	Total (N = 68)
Age, y	
18–21	0 (0)
22–25	36 (52.9%)
26–29	25 (36.8%)
30–33	5 (7.4%)
33 +	1 (1.5%)
Gender	
Male	35 (51.5%)
Female	32 (47.1%)
Prefer not to say	1 (1.5%)
Ethnicity	
Caucasian	33 (48.5%)
Black or African American	5 (7.4%)
Latinx or Hispanic	3 (4.4%)
Asian	14 (20.6%)
Native American	0 (0%)
Pacific Islander	4 (5.9%)
Two or More	7 (10.3%)
Prefer not to say	2 (2.9%)
Highest Degree Attained	
Bachelor	43 (63.2%)
Masters	24 (35.3%)
Doctorate	1 (1.5%)

### Motivations to Conducting Research

The majority of students [(39/68), 57.4%] feel that the USMLE Step 1 transition to pass/fail has influenced their attitudes and beliefs about conducting research in medical school. Twelve students (17.6%) did not answer this question. Students were then asked to select their level of agreement to a list of factors that play a role and/or motivate them to conduct research in medical school (Table 2). For these factors, responses of strongly agree and agree were grouped as “agree” for analysis. The majority of students indicated that they were motivated to conduct research to be more competitive for residency program applications [(65/68), 95.6%], to differentiate myself from other medical students [(56/68), 82.4%], and to be more involved in my interested specialty [(54/68), 79.4%]. This is consistent with the top three motivating factors students were asked to rank. Additionally, a majority of students agreed that gaining more knowledge in their specialty of interest [(42/68), 61.8%], interest in specific research topics [(40/68), 58.8%], and to advance the field [(39/68), 57.4%] are motivating factors. The difference in the motivating factors based on gender was not statistically different (all *p* > 0.05).

**Table 2:** Motivators

Factors that play a role/motivate students to conduct research	No. (%)				
	Strongly agree	Agree	Neutral	Disagree	Strongly disagree
To be more competitive for residency program applications	52 (76.5)	13 (19.1)	3 (4.4)	0 (0)	0 (0)
To differentiate myself from other medical students	33 (48.5)	23 (33.8)	8 (11.8)	2 (2.9)	2 (2.9)
To be more involved in my interested specialty	17 (25)	37 (54.4)	7 (10.3)	5 (7.4)	2 (2.9)
Interest in specific research topics	17 (25)	23 (33.8)	15 (22.1)	10 (14.7)	3 (4.4)
To gain more knowledge about my interested specialty	14 (20.6)	28 (41.2)	16 (23.5)	7 (10.3)	3 (4.4)
To advance the field	14 (20.6)	25 (36.8)	20 (29.4)	4 (5.9)	5 (7.4)
Interest in incorporating research into your medical career	9 (13.2)	18 (26.5)	19 (27.9)	13 (19.1)	9 (13.2)
To aid in clinical decision making	7 (10.3)	25 (36.8)	14 (20.6)	16 (23.5)	6 (8.8)

### Barriers to Conducting Research

Students were asked to select their level of agreement to which the stated factors served as barriers to conducting research in medical school (Table 3). For the factors listed, responses of strongly agree and agree were grouped as “agree” for analysis. The majority of students indicated that the barriers to conducting research are lack of time [(59/68), 86.8%], lack of mentoring [(43/68), 63.2%], lack of opportunities [(37/68), 54.4%], and lack of knowledge [(36/68), 52.9%]. The differences in factors that act as barriers based on gender were not statistically significant (all *p* > 0.05).

**Table 3:** Barriers

Factors that act as barriers to students conducting research	No. (%)				
	Strongly agree	Agree	Neutral	Disagree	Strongly disagree
Lack of time	24 (35.3)	35 (51.5)	6 (8.8)	3 (4.4)	0 (0)
Lack of mentoring	16 (23.5)	27 (39.7)	13 (19.1)	11 (16.2)	1 (1.5)
Lack of research opportunities	14 (20.6)	23 (33.8)	12 (17.6)	15 (22.1)	4 (5.9)
Lack of interest in research	13 (19.1)	20 (29.4)	10 (14.7)	19 (27.9)	6 (8.8)
Lack of funding	9 (13.2)	16 (23.5)	23 (33.8)	16 (23.5)	4 (5.9)
Difficulty obtaining approval for the study	7 (10.3)	16 (23.5)	29 (42.6)	12 (17.6)	4 (5.9)
Lack of knowledge	6 (8.8)	30 (44.1)	15 (22.1)	13 (19.1)	4 (5.9)
Research is not relevant to my preferred specialty	3 (4.4)	11 (16.2)	15 (22.1)	25 (36.8)	14 (20.6)

### Research Participation and Output

A majority of student respondents were actively participating in a research project [(49/68), 72.1%] than not [(19/68), 27.9%]. Students who have participated in research are involved in 1–2 research studies [(16/60), 26.7%], 3–5 research studies [(34/60), 56.7%], 6–10 research studies [(8/60), 13.3%], or 10 or more research studies [(2/60), 3.3%]. In terms of research output, as denoted by the number of abstracts, presentations, or articles published, students have produced 1–2 research products [(42/60), 70.0%], 3–5 research products [(9/60), 15.0%], 6–10 research products[(3/60), 5.0%], or 10 or more research products [(6/60), 10.0%]. There is a correlation between research participation and research output (*r* = 0.49, *p* < 0.001) among all students. Students who indicated that the Step 1 scoring change altered their attitudes about conducting research in medical school participated in significantly more studies than students who selected no (*p* = 0.006, ES = 0.21). However, among these students, there is no significant difference in research output (*p* = 0.59, ES = 0.03).

Students who perceived their interested specialty to be “not competitive” and “minimally competitive” were grouped, and students who perceived their interested specialty to be “moderately competitive” and “highly competitive” were grouped for analysis. There was a positive correlation between research participation and perception of specialty competitiveness (*r* = 0.36, *p* = 0.005). However, there was no correlation between research output and perception of specialty competitiveness (r = 0.12, *p* = 0.344).

The students were asked to select which specialties they were interested in pursuing. Students who indicated an interest in urology, general surgery, internal medicine, and otolaryngology (ENT) had significantly more research output than students who did not select those specialties (Table 4). There was no difference between genders in research participation (*p* = 0.58, ES = 0.08) and research output (*p* = 0.87, ES = 0.11).

**Table 4:** MSE specialty

# Research products from students who indicate the following specialties of interest	1–2 studies	3–5 studies	6–10 studies	> 10 studies	Rank bi serial-correlation coefficient	P-value
Anesthesiology	4	0	1	3	0.237	0.069
Child Neurology	3	0	0	0	-0.148	0.260
Dermatology	3	0	0	2	0.116	0.376
Diagnostic Radiology	4	1	0	2	0.128	0.330
Emergency Medicine	6	2	0	2	0.105	0.422
Family Medicine	6	1	1	0	-0.053	0.690
General Surgery	4	3	1	4	.429	0.001**
Internal Medicine (IM)	13	4	2	5	.314	0.015*
Combined IM and Pediatrics	0	1	0	0	0.154	0.242
Interventional Radiology	3	0	0	2	0.116	0.376
Neurosurgery	1	0	0	0	-0.084	0.525
Obstetrics and Gynecology	6	2	0	0	-0.074	0.576
Ophthalmology	1	1	0	0	0.050	0.706
Orthopedic Surgery	8	2	0	2	0.045	0.735
Otolaryngology	2	1	0	4	.395	0.002**
Pathology	1	0	0	0	-0.084	0.525
Pediatrics	8	5	1	0	0.093	0.480
Physical Medicine and Rehabilitation	8	1	0	2	-0.005	0.972
Plastic Surgery	1	0	0	2	0.246	0.058
Psychiatry	4	3	1	0	0.126	0.337
Radiation Oncology	2	1	0	2	0.226	0.082
Urology	1	1	0	2	.294	0.023*
Vascular Surgery	1	0	0	1	0.119	0.363

*p ≤ 0.05, **p ≤ 0.01

### Open-Ended Responses

An open-ended survey question captured forty student comments on how they envision research playing a role in their future career. 15 students indicated a future role of research to advance medical knowledge and patient care. Students wrote that research would help in “advancing and developing current knowledge to better address patient care needs” and “incorporating [the] latest technology and knowledge to patient care. Aiding ongoing research initiatives.” Furthermore, four students reported a role of research in their future careers in academic medicine. One student wrote, “I am interested in academic medicine and would like to incorporate research by also directly mentoring students, but it would not be a huge priority.”

Conversely, nine students indicated that they prioritize patient and clinical work as their primary focus with research as an adjunct pursuit. One student wrote, “I hope to participate and engage with research but I am becoming a physician to treat patients” and another student wrote, “adjunct to clinical practice.” Similarly, another student wrote, “I would like to conduct research, but secondary to my role as a clinician.” In contrast, 11 students responded that they do not envision a role of research in their future careers. One student wrote, “I do not plan on doing research in my future career,” and another wrote, “I don’t see research play a role in my future career. I hope to work purely in a clinical setting.” Another five students were unsure of the role of research in their future career, stating, “Honestly, I’m not certain…. it depends on what I choose to do in the future, and whether I feel driven to continue research.”

## Discussion

We surveyed second-year medical students about their attitudes and beliefs about conducting research during medical school. The top motivating factors for medical students participating in research during medical school are to be more competitive for residency and to differentiate themselves from other students. These factors suggest that medical students believe that research participation is an increasingly important differentiating factor in competitive residency applications. This belief aligns with the finding from Wolfson et. al. during their survey of residency program directors that highly competitive residency programs use research participation as a differentiating factor in their evaluations [6, 7]. Though students are participating in research projects to be more competitive for residency applications, the majority of students in this study agreed that participating in research was a good way to get more involved, gain more knowledge, and explore specific topics of interest in their preferred specialty. This result may reflect a belief that research participation provides opportunities to network, explore the specialty beyond what is presented during didactics and clerkship rotations, and demonstrate early commitment to the specialty, something program directors cite as important to see in candidates [3].

The top barrier to research participation for medical students is lack of time. Research has a steep learning curve and each step from project conception to submitting an IRB to recruiting a sizable participant cohort to data collection and writeup, submission, peer review, and feedback takes time [14]. Students hoping to match into competitive specialties are electing to take a research year to have extra time to build their research portfolio [15]. Some schools moving to a 3-year curriculum have carved out an extra year during which students can participate in research or extra-curricular activities but still graduate within 4-years [16]. This is in contrast to existing literature on medical student cited barriers, which reports lack of funding and opportunities[17] to be the topmost cited barrier. Our study does reflect these sentiments in eliciting the lack of mentoring and research opportunities as the next most cited barrier. These barriers may reflect a limited research ecosystem and infrastructure specific to our institution. Due to a lack of resources at the home institution, medical students are increasingly traveling to outside institutions or reaching out to their contacts to increase competitiveness, which builds upon an inherent disparity among their peers without the means or the network [18].

Findings of our study demonstrate research output to be positively correlated with research participation. Research is largely a trial-and-error process, and students may get involved in multiple research projects to maximize the likelihood that at least a few of them will be productive. Between research participation and research output, we found that only research participation was significantly correlated with perceived specialty competitiveness. Students were administered this questionnaire relatively early in their medical school career and their research output may not reflect their higher participation. When we distilled competitiveness to individual specialties, our analysis indicated that students interested in Internal Medicine (IM), General Surgery, Otolaryngology and Urology had significantly higher research output than the students who did not select those specialties. This result likely reflects the level of engagement of students with these specialty departments, such as an active student-run interest group, the capacity for medical students to be involved in research projects in that department, and most importantly, the presence of a home residency program that provides opportunities for shadowing, networking, and research. Internal Medicine and General Surgery also have established residency programs at EVMS, but their significance also likely encapsulates the dichotomy of students who are leaning towards either medicine or surgery and were still relatively undifferentiated at the time of the survey.

We compared the motivators, barriers, research participation, output, and competitiveness by gender and no measure was significantly different between male and female-identifying students. This is in contrast to a previous study that reported female students perceiving more barriers to medical research [19]. The lack of an overt gender disparity may reflect a national trend of women in medicine [20]. It may also be due to the presence of active student interest groups catering to female medical students such as the EVMS chapter of The Association of Women Surgeons.

Finally, we note that many students recognize a role for research in medicine. Qualitative analysis of the open-ended question elicits further motivations for students’ involvement in research, including to advance their field, to align with their pursuit of academic medicine, and as an adjunct to their clinical practice. These sentiments are consistent with the widely held attitude that research is the currency of academia [21]. Interestingly, a study conducted by Sanabria-de la Torre challenges the adjunctive role of research within clinical practice by asserting that as students dedicate themselves more to clinical practice, they lose their interest in scientific research [17]. There was a minority of students who reported no role or were unsure of the role of research in their future career. This sentiment may reflect a narrow concept of research rather than a set of critical thinking and communication skills that will continue to be relevant beyond school and residency applications, in alignment with the study from Cornett et. al. about scholarly endeavors building research skills [22].

Altogether, our study distills a set of recommendations for institutions in supporting medical students in their academic careers. The low-hanging fruit is to address the barriers, ie, lack of time, mentorship, and research opportunities. Restructuring of the medical school curriculum to carve out as much as a year’s length of dedicated research time while still being able to graduate in four years is a durable solution that aligns with the pass/fail scoring system of Step 1. Medical schools without robust research ecosystems may choose to partner with nearby undergraduate institutions with a robust research department. Additionally, a lot of scholarly work can be conducted on large clinical datasets, access to which medical schools can purchase for students. Research infrastructure does not only come from research laboratories and available PIs, but also from IRB, funding, etc. Having dedicated personnel to carry the burden of IRB proposal writing would also be a large onus relieved from the medical student. The finding that students aligned with specialties with home programs was unanticipated but not surprising. The clinical staff, ie, attendings and residents, are the most well-positioned for clinical research. Medical students should see their home residency programs as an important resource, not only for career advising but for research opportunities, and medical schools should work to promote this relationship, not only at the clerkship phase but pre-clerkship, when students have more time and energy to engage in scholarly work. Lastly, the finding that few students see research as an integral part of their future clinical career might encourage medical institutions to shape students’ long-term outlook on research as integral to patient care by incorporating landmark clinical trials during lectures that shaped medical algorithms today.

There are a few limitations to this study. This is a cross-sectional study at one university; therefore, the results may not be generalizable. Additionally, the recruitment process may introduce selection bias. Our survey relied on voluntary participation, and students who have strong opinions about the research process may be more motivated to complete the survey. This was mitigated by communication about the survey from a relatively neutral source who is not involved in clinical duties or residency programs. Finally, the data is self-reported, which may not match actual research participation and output.

## Conclusion

Second-year medical students at a single institution following a traditional two-year preclinical curriculum perceive research participation and productivity to be an increasingly important differentiating factor in competitive residency applications as the USMLE Step 1 transitions to pass/fail. Students have also cited a lack of time, mentorship, and opportunities to pose the greatest barriers to research participation. No significant differences exist between genders on motivators, barriers, and research participation. Students recognize the role of research productivity in residency applications. However, based on student comments, a minority of students positively indicated participating in research in their future clinical careers. These findings suggest that supporting student endeavors in academic or clinical research is a crucial aspect of a medical school curriculum. Specifically, these efforts could focus on creating more opportunities for student participation and matching students with mentors, especially in specialties for which the institution does not have a residency program.

## Supplementary Information

Below is the link to the electronic supplementary material.

Supplementary file1 (PDF 45.0 KB)

## Data Availability

The datasets used and/or analyzed during the current study are available from the corresponding author upon reasonable request.

## Abbreviations

EVMS: Eastern Virginia Medical School
NRMP: National Residency Match Program
