# Supplementary material for: Medical Students’ Attitudes Towards Research Participation After the United States Medical Licensing Examination Step 1 Scoring Change
Source: Med Sci Educ. 2025 Sep 19;35(6):2995–3002. doi: 10.1007/s40670-025-02508-3 (PMC12960994; doi:10.1007/s40670-025-02508-3)
Supplement: Supplementary file 1 — Supplementary file1 (PDF 45.0 KB) [file 40670_2025_2508_MOESM1_ESM.pdf]

Medical Student's Attitudes and Motivations Towards Research Survey

Please complete the survey below.

Thank you!

---

1) Do you feel that the USMLE STEP-1 transition to pass/fail has influenced your attitudes and beliefs about conducting research in medical school?

- ◯ Yes
- ◯ No

Please select your level of agreement to which the stated factors play a role/motivate you to conduct research in medical school:

		1. Strongly Agree	2. Agree	3. Neutral	4. Disagree	5. Strongly Disagree
2)	Interest in incorporating research into your medical career	◯	◯	◯	◯	◯
3)	To gain more knowledge about my interested specialty	◯	◯	◯	◯	◯
4)	To be more involved in my interested specialty	◯	◯	◯	◯	◯
5)	Interest in specific research topics	◯	◯	◯	◯	◯
6)	To differentiate myself from other medical students	◯	◯	◯	◯	◯
7)	To be more competitive for residency program applications	◯	◯	◯	◯	◯
8)	To aid in clinical decision making	◯	◯	◯	◯	◯
9)	To advance the field	◯	◯	◯	◯	◯

---

10) Of the factors stated, pick the top 3 that has the most influence in you conducting research in medical school:

- ☐ Interest in incorporating research into my medical career
- ☐ To gain more knowledge about my interested specialty
- ☐ To be more involved in my interested specialty
- ☐ Interest in specific research topics
- ☐ To differentiate myself from other medical students
- ☐ To be competitive for residency program applications
- ☐ To aid in clinical decision making
- ☐ To advance the field

---

11) If you have another motivating factor/s that are not listed, please write it down:

____

Please select the level of agreement to which the stated factors act as barriers to you conducting research in medical school:

		1. Strongly Agree	2. Agree	3. Neutral	4. Disagree	5. Strongly Disagree
12)	Lack of interest in research	○	○	○	○	○
13)	Research is not relevant to my preferred specialty	○	○	○	○	○
14)	Lack of knowledge	○	○	○	○	○
15)	Lack of research opportunities	○	○	○	○	○
16)	Lack of time	○	○	○	○	○
17)	Lack of mentoring	○	○	○	○	○
18)	Lack of funding	○	○	○	○	○
19)	Difficulty obtaining approval for the study	○	○	○	○	○

20) Of the factors stated, pick the top 3 factors that act as barriers to you conducting research in medical school?

- ☐ Lack of interest in research
- ☐ Research is not relevant to my preferred specialty
- ☐ Lack of knowledge
- ☐ Lack of research opportunities
- ☐ Lack of time
- ☐ Lack of mentorship
- ☐ Lack of funding
- ☐ Difficulty obtaining approval for the study

21) If you have other barrier/s that are not listed, please write it down:

____

22) Have you been involved in research before medical school?

- ○ Yes
- ○ No

23) Are you currently working on a research project?

- ○ Yes
- ○ No

24) If yes, how many research studies have you participated in?

- ○ 1-2 research studies
- ○ 3-5 research studies
- ○ 6-10 research studies
- ○ 10 or more research studies

25) How many abstracts/presentations/articles have you published?

- ○ 1-2 research studies
- ○ 3-5 research studies
- ○ 6-10 research studies
- ○ 10 or more research studies

26) How would you describe the competitiveness of the specialties you are interested in pursuing?

- ○ Not competitive
- ○ Minimally competitive
- ○ Moderately competitive
- ○ Highly competitive

27) What specialties are you interested in pursuing?

- ☐ Anesthesiology
- ☐ Child Neurology
- ☐ Dermatology
- ☐ Diagnostic Radiology
- ☐ Emergency Medicine
- ☐ Family Medicine
- ☐ General Surgery
- ☐ Internal Medicine
- ☐ Internal Medicine/Pediatrics
- ☐ Interventional Radiology
- ☐ Neurosurgery
- ☐ Obstetrics and Gynecology
- ☐ Ophthalmology
- ☐ Orthopedic Surgery
- ☐ Otolaryngology
- ☐ Pathology
- ☐ Pediatrics
- ☐ Physical Medicine and Rehabilitation
- ☐ Plastic Surgery
- ☐ Psychiatry
- ☐ Radiation Oncology
- ☐ Urology
- ☐ Vascular Surgery

28) Describe the role of research in your future career:

____

29) What gender do you identify as?

- ◯ Male
- ◯ Female
- ◯ Other
- ◯ Prefer not to say

30) What is your age range?

- ◯ 18-21
- ◯ 22-25
- ◯ 26-29
- ◯ 30-33
- ◯ 33+

31) Please specify your ethnicity.

- ◯ Caucasian
- ◯ Black or African-American
- ◯ Latinx or Hispanic
- ◯ Asian
- ◯ Native American
- ◯ Native Hawaiian or Pacific Islander
- ◯ Two or More
- ◯ Other/Unknown
- ◯ Prefer not to say

32) What is your highest degree received?

- ◯ Bachelor Degree
- ◯ Masters Degree
- ◯ Doctorate Degree
